# PP53 Analysis Of Knee Arthroplasty Registries Use In Developing Scientific Evidence: A Scope Review

**DOI:** 10.1017/S0266462325102729

**Published:** 2025-12-29

**Authors:** Cristiane Rocha de Oliveira, Roberto Carlos Lyra Silva, Grasiela Martins da Silva, Quenia Cristina Dias Morais e Verônica Clemente

## Abstract

**Introduction:**

Although randomized controlled trials (RCTs) are the gold standard for evaluating health interventions, their methodology is challenging for arthroplasties. An international network of arthroplasty registries (AR) provides real-world data to generate evidence. However, the extent to which AR data contribute to scientific evidence remains unclear. This scoping review aimed to analyze the use of AR data in scientific publications.

**Methods:**

All methodological stages of a scoping review were conducted in accordance with the Joanna Briggs Institute manual. The database search utilized official Medical Subject Heading and Health Science Descriptors terms, retrieving 25,846 studies. Study selection was performed in two stages by pairs of reviewers to ensure accuracy and reliability. The report followed the PRISMA Extension for Scoping Reviews. The analysis of the results was carried out using descriptive categories to classify and interpret the data comprehensively. No funding was provided for this study.

**Results:**

After selection, 140 studies were included. Studies on national AR were most prevalent in Australia and the UK, followed by studies on institutional and regional AR in the USA. Retrospective cohort designs dominated, involving large and heterogeneous populations. Total and unicompartmental arthroplasties were the primary subjects, with surgical revision being the most reported outcome. Authors identified limitations related to AR data application and observational study methodologies, impacting evidence reliability.

**Conclusions:**

When properly implemented, AR are crucial data repositories that enable the generation of scientific evidence on arthroplasty technologies, predictive failure models, and post-sale prosthesis monitoring. This demands institutional efforts, collaborations, and researcher training for the proper use of observational studies, alongside paradigm shifts in confidence toward the evidence produced to support decision-making.

